# Association of *PER2* Genotype and Stressful Life Events with Alcohol Drinking in Young Adults

**DOI:** 10.1371/journal.pone.0059136

**Published:** 2013-03-22

**Authors:** Dorothea Blomeyer, Arlette F. Buchmann, Jesus Lascorz, Ulrich S. Zimmermann, Günter Esser, Sylvane Desrivieres, Martin H. Schmidt, Tobias Banaschewski, Gunter Schumann, Manfred Laucht

**Affiliations:** 1 Department of Child and Adolescent Psychiatry and Psychotherapy, Central Institute of Mental Health, Medical Faculty Mannheim, University of Heidelberg, Mannheim, Germany; 2 Division of Molecular Genetic Epidemiology, German Cancer Research Center (DKFZ), Heidelberg, Germany; 3 Department of Psychiatry and Psychotherapy, University Hospital Carl Gustav Carus, Technical University, Dresden, Germany; 4 Department of Psychology, University of Potsdam, Potsdam, Germany; 5 Social, Genetic & Developmental Psychiatry Centre (MRC), Institute of Psychiatry, King’s College, London, United Kingdom; Centre for Addiction and Mental Health, Canada

## Abstract

**Background:**

Clock genes govern circadian rhythms and shape the effect of alcohol use on the physiological system. Exposure to severe negative life events is related to both heavy drinking and disturbed circadian rhythmicity. The aim of this study was 1) to extend previous findings suggesting an association of a haplotype tagging single nucleotide polymorphism of *PER2* gene with drinking patterns, and 2) to examine a possible role for an interaction of this gene with life stress in hazardous drinking.

**Methods:**

Data were collected as part of an epidemiological cohort study on the outcome of early risk factors followed since birth. At age 19 years, 268 young adults (126 males, 142 females) were genotyped for *PER2* rs56013859 and were administered a 45-day alcohol timeline follow-back interview and the Alcohol Use Disorders Identification Test (AUDIT). Life stress was assessed as the number of severe negative life events during the past four years reported in a questionnaire and validated by interview.

**Results:**

Individuals with the minor G allele of rs56013859 were found to be less engaged in alcohol use, drinking at only 72% of the days compared to homozygotes for the major A allele. Moreover, among regular drinkers, a gene x environment interaction emerged (p = .020). While no effects of genotype appeared under conditions of low stress, carriers of the G allele exhibited less hazardous drinking than those homozygous for the A allele when exposed to high stress.

**Conclusions:**

These findings may suggest a role of the circadian rhythm gene *PER2* in both the drinking patterns of young adults and in moderating the impact of severe life stress on hazardous drinking in experienced alcohol users. However, in light of the likely burden of multiple tests, the nature of the measures used and the nominal evidence of interaction, replication is needed before drawing firm conclusions.

## Introduction

Alcohol use disorders (AUDs) are regarded as one of the most severe public health problems worldwide, being the leading cause of a wide variety of morbidity and mortality conditions in many developed countries. Research has amply demonstrated that both genetic and environmental factors contribute to the development of these disorders [1]–[5]. The psychoactive effects of alcohol are mediated through its influence on different functional systems in the brain, including the dopamine, serotonin, gamma-aminobutyric acid, glutamate, and opioid peptide systems. Another system whose involvement in the etiology and continuation of alcohol abuse has recently been proposed is the circadian clock mechanism [6]. The circadian clock optimizes the adaptation of an organism to its internal and external environment. In animal models, the time of day was demonstrated to shape the rewarding effect of substance use [7], [8]. In turn, circadian rhythmicity was found to be influenced by substance use, in general, and alcohol use, in particular, notably heavy use [9]. This becomes apparent in severe disorganizations of physiological clock systems, such as sleep, body temperature or blood pressure during chronic alcohol abuse, dependence and withdrawal, with these disorganizations being based on altered expression of the clock genes.

One of the clock genes involved is the *PER2* gene. *PER2* has been associated with behavioral adaptation to environmental stressors in humans [10] and to enhanced alcohol intake in *PER2* mutant mice [11]. In an exploratory analysis of the human *PER2* gene, the latter researchers reported first evidence for an association of allelic variation in *PER2* gene with drinking levels in humans, which was driven by single nucleotide polymorphism (SNP) rs56013859 carrying an A/G exchange in intron 3 at position 1071 bp downstream of the ATG site of *PER2* (GI:1365471).

Severe stressful experiences, such as the death of a loved one, break-up of a relationship, or job loss usually go along with a destabilization of living conditions and an elevated level of unpleasant feelings. In such changing life situations, alcohol use, particularly heavy use, can reduce negative feelings and, in the beginning, dampen unpleasant physiological phenomena, such as sleeplessness or restlessness [2]. Therefore, drinking is frequently used as a means to cope with stress [2], [12]–[14]. However, growing tolerance towards the effects of alcohol can lead to increased amounts being drunk, thereby aggravating life stress and altering circadian rhythmicity [15]. Accordingly, life stress is regarded as a major environmental risk factor for both, heavy drinking [16], [17] and disturbed circadian rhythmicity. Possible explanations for this link include biological mechanisms, by which alcohol use alters the physiological reaction to stress, namely the hypothalamic-pituitary-axis response, or vice versa [4], [17], [18].

Adolescence and young adulthood are periods of particular importance for the development of substance use disorders, as the brain is still in a developing phase and thus prone to alcohol related alterations [19], [20]. In half of the cases, AUDs manifest by the age of 23, with the highest incidence between 18 and 23 years [21], [22]. On the behavioral level, the rate of heavy drinking decreases steeply in most people after young adulthood, but a high risk group maintains this hazardous drinking pattern and develops alcohol use disorders [23]. These results highlight the importance of early drinking patterns for the development of AUDs. In a sample which has not yet reached the age of the peak incidence of AUDs, it is of special interest to investigate drinking patterns prone to or indicative of these disorders.

Given the role of clock genes and stress in heavy alcohol use, the aim of the present study was twofold: 1) to extend previous findings suggesting an association of *PER2* rs56013859 genotype with drinking patterns in an epidemiological cohort study of young adults from a high-risk sample, and 2) to examine a role for this genotype in moderating the effect of exposure to severe stressful life events on hazardous drinking. To consider a possible impact of drinking to cope with stress, only experienced alcohol consumers (i.e. with current regular use) were included in the latter analysis.

## Materials and Methods

### Ethics Statement

The study was approved by the ethics committee of the University of Heidelberg and written informed consent was obtained from all participants.

### Participants

Data were collected as part of the Mannheim Study of Children at Risk, an ongoing epidemiological cohort study of the outcome of early risk factors from infancy into adulthood [24]. The initial sample comprised 384 children of predominantly (>99.0%) European descent, born between 1986–88. Infants were recruited from two obstetric and six children’s hospitals of the Rhine-Neckar Region of Germany and were included consecutively into the sample according to a two-factorial design intended to enrich and control the status of the sample regarding obstetric and psychosocial risks (full details of the sampling procedure have been reported previously) [25]. Only firstborn children with singleton births and German-speaking parents were enrolled in the study. As well, children with severe physical handicaps, obvious genetic defects, or metabolic diseases were excluded. Assessments were conducted at regular intervals throughout development, most recently at the age of 19. Of the initial sample of 384 participants, 18 (4.7%) were excluded because of severe handicaps (IQ<70 or neurological disorder), 39 (10.2%) were dropouts, 35 (9.1%) refused to participate in blood sampling and 24 (6.3%) had incomplete data. Accordingly, the current investigation included 268 young adults (126 males, 142 females) for whom data on *PER2* genotype, stressful life events and alcohol use at age 19 were available. Among these were 131 current regular alcohol users (80 males, 51 females), defined as drinking at least once per week. Loss of subjects was not selective with regard to obstetric or psychosocial risks.

### Assessment

A 45-day timeline follow-back (TLFB) interview [26] was administered to assess *current drinking behavior* in young adults, providing estimates of the distribution of drinking days and the amount of daily alcohol consumption. To assist recall, participants were asked to bring their time planner to the interview. Aided by the diary, the interviewer then inquired about the number of drinks on each day, beginning with the current day and working backward. In the literature, there is ample evidence of the reliability and validity of the TLFB method [27]. The number of drinking days and the total number of drinks were derived. Individuals with 6 or more drinking days in the last 45 days (i.e. with approximately a weekly intake) were assigned to the regular alcohol user group.

Additionally, the young adults completed the Alcohol Use Disorders Identification Test (AUDIT), a screening instrument for the detection of hazardous alcohol use, developed by the WHO [28]. The AUDIT comprises 10 items, referring to the last 12 months, by which patterns of alcohol consumption (items #1–3), alcohol dependence (items #4–6) and adverse consequences of heavy drinking (items #7–10) are assessed. The AUDIT has shown reasonable reliability and validity in a German sample [29]. The score is considered as a continuum from abstaining to harmful drinking to alcohol dependence [30]. Theoretically, AUDIT values up to 40 are possible; the scores in this sample varied from 0 to 21.

Stressful life events were assessed with a modified and shortened version of the Munich Events List (MEL) [31]. The questionnaire asked for occurrence and threat of severe life events and chronic difficulties in the four years prior to the assessment in young adulthood. The items addressed all areas of a young adult’s life from school to job, partner, family, parents, living conditions, legal troubles, up to health problems. Threat was rated on a scale ranging from 1 (not stressful) to 5 (very heavy stress). The total score of acute severe life events was calculated as the sum of 38 items, referring to events with acutely stressful impact, such as the death of a loved one or relationship breakup, which rated at a stress level of 3 (considerably stressful) or more. In this sample, the score ranged from 0 to 15 events (M = 2.86, SD = 2.60). Psychometric characteristics of the MEL have been confirmed in several studies [32].

### Genotype Analysis

DNA was prepared from whole blood with standard salting out methods. *PER2* rs56013859 was genotyped using the TaqMan MGB biallelic discrimination system. Probes and primers were ordered from and automatically designed by Applied Biosystems using the Assay-by-Design product. PCR reactions were performed in Biometra T1 thermocyclers, and fluorescence results were determined with the use of an ABI Prism 7900HT sequence-detector end-point read. Process and genotyping data were exported into an internal LIM System. Distribution of rs56013859 genotypes was AA = 78.3%, AG = 19.6%, and GG = 2.1% in accordance with Hardy-Weinberg equilibrium (p = .254).

### Data Analysis

Differences according to *PER2* genotype and sex were examined using a two-way (*PER2* × sex) ANOVA. To assess the effects of genotype, stressful life events and their interaction on alcohol use in regular drinkers, a linear regression model was performed. After adjusting for the main effects of *PER2* and stressful life events, the gene x environment effect was tested as additional interaction term. Significant interactions were further investigated using simple main effects analyses. Sex was included as a covariate. For these analyses, *PER2* rs56013859 was classified according to homozygosity for the major A allele (i.e., dominant model for the minor G allele). Stressful life events were entered as a continuous variable. The current sample of N = 131 regular drinkers has a power to detect interaction effects of small effect size according to Cohen (i.e., f^2^ = .02) with p<.05 of 36.2% and of medium effect size (i.e., f^2^ = .15) of 99.3% [33].

## Results

Table 1 displays descriptive statistics for the total sample and by genotype groups, adjusted for sex. Bivariate comparisons revealed no significant sex differences with regard to family adversity, obstetric complications, and negative life events, with the exception that females were somewhat older than males. Sex had an effect on all drinking measures, indicating that males drank more hazardously, had more drinking days and consumed a higher total number of drinks than females (data not shown). The genotype groups did not differ on any of these variables. However, consistent differences according to genotype were observed for the drinking measures. Carriers of the minor G allele had less drinking days than homozygotes for the A allele and, as a trend, consumed a lower number of drinks. Similar results with regard to control variables, life events and drinking measures were obtained in the subgroup of regular alcohol users (Table 1).

**Table 1:** Demographic and clinical characteristics of young adults by *PER2* rs56013859 genotype: means and SE (in parenthesis) adjusted for sex.

Total sample	rs56013859 genotype			Total
	AA	AG/GG	p	
	(n = 209)	(n = 59)	(difference)	(n = 268)
Age (years)	19.2 (0.2)	19.3 (0.4)	.282	19.2 (0.25)
Family adversity score^1^	1.98 (0.15)	1.85 (0.27)	.667	1.91 (0.15)
Obstetric risk score^2^	1.09 (0.07)	1.02 (0.14)	.633	1.05 (0.08)
Severe negative life events	2.74 (0.17)	2.24 (0.32)	.165	2.49 (0.18)
Number of drinking days^3^	7.53 (0.45)	5.37 (0.84)	**.025**	6.45 (0.48)
Total number of drinks^3,4^	30.9 (2.7)	21.1 (5.1)	.*094*	26.0 (2.9)
AUDIT score	4.95 (0.25)	4.16 (0.46)	.133	4.55 (0.26)

Subsample of regular drinkers	AA	AG/GG	p	
	(n = 107)	(n = 24)	(difference)	(n = 131)
Age (years)	19.2 (0.3)	19.3 (0.6)	.026	19.3 (0.04)
Family adversity score^1^	1.97 (0.21)	2.08 (0.44)	.833	2.03 (0.24)
Obstetric risk score^2^	1.03 (0.09)	1.05 (0.19)	.920	1.04 (0.11)
Severe negative life events	2.95 (0.26)	2.42 (0.55)	.389	2.69 (0.61)
Number of drinking days^3^	12.64 (0.61)	9.76 (1.28)	**.044**	11.20 (0.71)
Total number of drinks^3,4^	54.7 (4.6)	43.3 (9.7)	.286	49.0 (5.4)
AUDIT score	6.62 (0.36)	5.97 (0.77)	.443	6.30 (0.42)

1 “Enriched” family adversity index as proposed by Rutter and Quinton (1977) measuring the presence of 11 adverse family factors covering characteristics of the parents, the partnership, and the family environment during a period of one year prior to birth;

2 obstetric adversity score counting the presence of 9 adverse conditions during pregnancy, delivery, and postnatal period such as preterm labor, asphyxia or seizures;

3 referring to the last 45 days;

4 number of standard drinks, each containing 10–13 g alcohol.

The results of linear regression analyses testing for the effects of *PER2* rs56013859 genotype, exposure to severe negative life events and the interaction thereof on drinking measures among regular drinkers are presented in Table 2. Analyses revealed a main effect of genotype on the number of drinking days within the past 45 days with G allele carriers consuming alcohol 2.990 (SE = 1.57) days less frequently than A homozygotes. In addition, there was a main effect of severe negative life events on both the total number of drinks used and the AUDIT score, indicating that hazardous alcohol use increased with the number of stressful experiences. Specifically, each additional life event increased the amount of alcohol consumed during the past 45 days by 6.506 (SE = 0.572) drinks and the AUDIT score by 0.572 (SE = 0.112).

**Table 2:** Linear regression models testing the effects of *PER2* rs56013859 genotype, severe negative life events and their interaction on drinking measures in young adult regular drinkers (n = 131).

Drinking measures^1^	rs56013859 genotype		Severe negative life events		rs56013859 × severe negative life events	
	B	p	B	p	B	p
Number of drinking days^2^	−2.929 (1.450)	**.046**	0.276 (0.205)	.181	0.110 (0.628)	.861
Total number of drinks^2^	−9.871 (10.215)	.336	6.506 (1.443)	**<.001**	−2.704 (4.416)	.541
AUDIT score	−0.437 (0.795)	.584	0.572 (0.112)	**<.001**	−0.786 (0.337)	**.021**

Note: All models adjusted for sex and age. Main effects of genotype and severe negative life events were entered in a first step, followed by the interaction term in a second step.

1 Unstandardized regression coefficients from linear regression (standard errors);

2 referring to the last 45 days.

**Figure 1:**
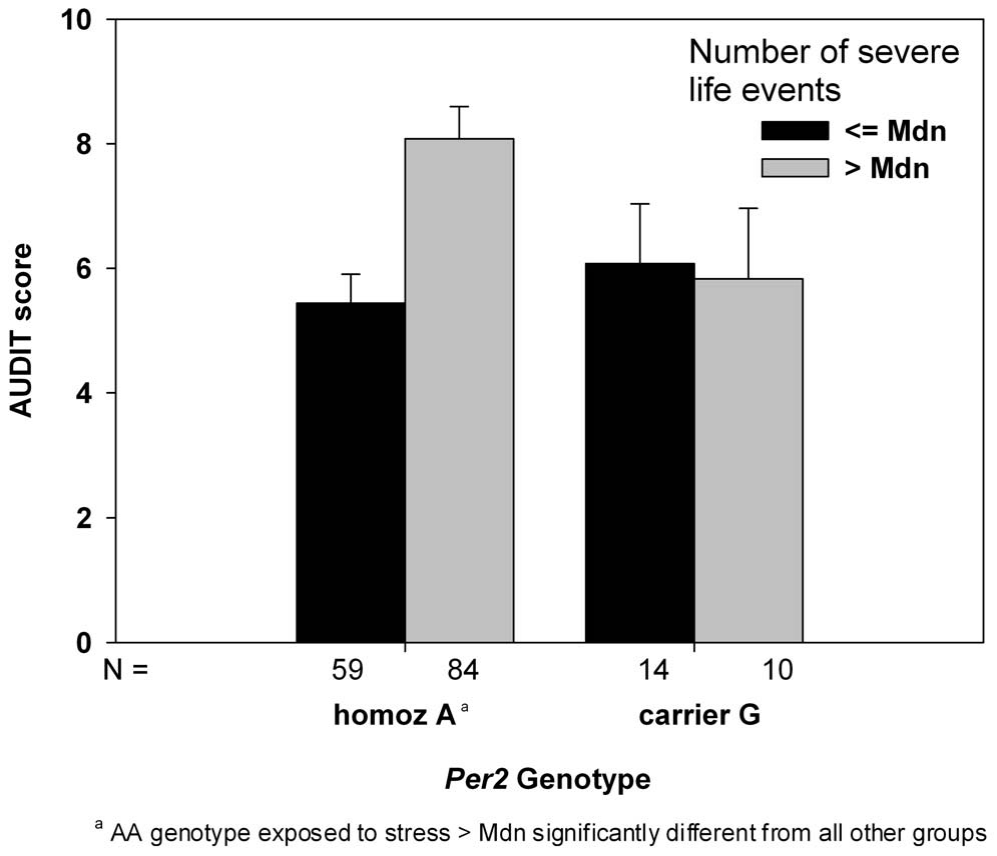
Mean AUDIT scores (SE), adjusted for sex, in young adults grouped by *PER2* rs56013859 genotype and exposure to negative life events.

Moreover, an interaction between *PER2* genotype and exposure to severe life stress on the AUDIT score emerged. Subsequent simple regression analyses stratified for genotype demonstrated that the number of severe negative life events was associated with higher scores on the AUDIT among individuals homozygous for the A allele (B = .669, *p*<.001), but not among carriers of the G allele (B = −.108, *p = *.674). Specifically, in A homozygotes, each additional life event increased the AUDIT score by 0.669 (SE = 0.123), while not significantly decreasing the AUDIT score by 0.108 (SE = 0.268) in G carriers. Figure 1 visualizes the interaction, using a median split on the life events measure. There were no other interactions between *PER2* genotype and severe life stress with regard to further drinking measures.

## Discussion

The present study provides further evidence, suggesting a possible role for allelic variation of the *PER2* gene in alcohol drinking in humans. In agreement with previous exploratory findings by Spanagel et al. [11], this study indicates that carriers of the G allele of a *PER2* haplotype tagging single nucleotide polymorphism (SNP rs56013859) were less engaged in alcohol drinking than individuals homozygous for the A allele. As the G allele is the minor allele of this polymorphism, this finding can be interpreted as a protective effect of the G allele on the susceptibility to alcohol abuse. While Spanagel et al. observed an association of this SNP with high vs. low alcohol intake in a group of alcohol-dependent patients, this study confirmed the association in young adults from a high-risk community sample.

The current investigation extends the findings of Spanagel et al. [6] by demonstrating that, among experienced alcohol users in a nonclinical sample, variation in the *PER2* gene may moderate the impact of stress on hazardous or harmful drinking. Our results revealed that exposure to stressful life events during the past four years was associated with higher AUDIT scores in homozygotes for the A allele of SNP rs56013859, but was unrelated among individuals carrying the G allele of this SNP. The AUDIT test is used to detect the preliminary signs of hazardous drinking and mild dependence, being one of the most accurate alcohol screening tests available [28]. Interestingly, this gene-stress interaction was restricted to signs of harmful use and did not refer to patterns of drinking, such as frequency or amount of alcohol use. This finding that the *PER2* genotype was associated with patterns of drinking behavior in both experienced and inexperienced alcohol users and with signs of hazardous use following stress in experienced users may be interpreted as suggesting a role of this gene in both the initiation and the progression of use.

Adolescence and early adulthood are periods of life which can become increasingly stressful. Such increases in exposure to stress may account for the rise in prevalence rates of alcohol intake during these age periods, in general, and of stress-reactive drinking, in particular [34], [35]. Overall, our findings indicated that the level of stress during the past four years was associated with higher current drinking. This association was most marked regarding the AUDIT score (i.e. signs of harmful drinking), while the number of drinking days appeared to be unrelated to stress.

**Figure 2:**
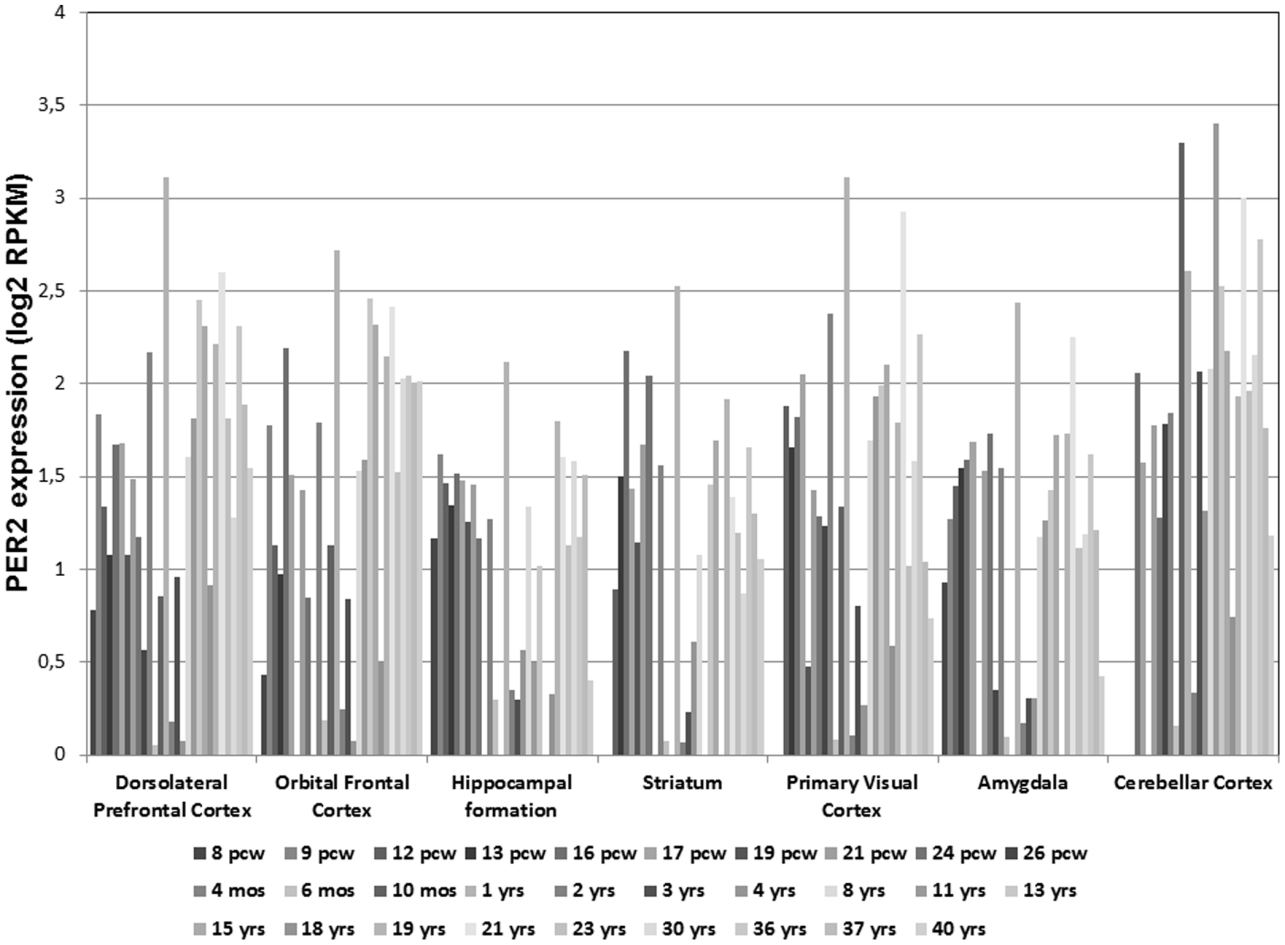
Spatiotemporal mRNA expression patterns of *PER2* in humans. Data were extracted from Brain Span, Atlas of Developing Human Brain.

Interest in *PER2* as a candidate gene for alcohol use resulted from animal studies [11]. Earlier experiments in rodents have shown that genetic variation in *PER2* is associated with a hyperglutamatergic state, which in turn leads to a higher level of alcohol use. The pharmaceutical drug acamprosate is supposed to act on this mechanism in order to reduce stress-induced craving. The present investigation provides no evidence for the exact mechanism by which the specific *PER2* genotype studied protects against risk of alcohol problems. In particular, our findings leave open the question as to how exposure to stress influences pathophysiological pathways by which the gene-environment interaction observed in our study is mediated. SNP rs56013859 is localized in the third intron of the Per2 gene and is not in linkage disequilibrium (LD) with common variants analyzed in Hapmap. Hypotheses on the functional role of variation in *PER2* suggest that SNP rs56013859 may alter the binding motives for transcription factors Sp1, c-myb, and nuclear factor B, suggesting a possible regulatory function of this SNP in transcriptional activation of *PER2* [11]. However, one limitation of this study is that we did not find any allele-specific expression of *Per2* in peripheral blood from IMAGEN participants or on publicly available eQTL browsers of cortical tissue. Since there is distinct temporal variation and pronounced expression differences of *Per2* across the life span (Figure 2) indicating temporal and regional-specific regulatory mechanisms, these results do not rule out a role of SNP rs56013859 or perhaps rare or intermediate variants in linkage disequilibrium with this SNP in transcriptional regulation. Recently, Agapito et al. [36] demonstrated a *PER2* mutation in mice to be associated with the regulation of β-endorphin release to acute and chronic ethanol challenges, providing another clue to the mechanism of different effectiveness of alcohol drinking as a means to cope with life events.

Our findings add to the growing knowledge implicating genotypic variation in the moderation of the individual’s response to stress exposure [10], [37]–[39]. In particular, they parallel evidence from our study demonstrating a moderating effect of the *CRHR1* genotype on the relationship between stressful life events and drinking behavior in adolescents [40]. In 15-year-olds, the number of negative life events during the past three years was found to be related to increasing rates of heavy drinking only among individuals carrying a specific genotype of a haplotype tagging SNP of this gene. While activation of brain circuits involved in stress regulation is considered as the biological basis of the latter gene-environment interaction, a different physiological mechanism has been suggested to underlie the *PER2* x stress interaction. The *PER* genes (*PER1, PER2* and *PER3*) in general, together with other clock genes, are part of the central circadian rhythm organization system in the suprachiasmatic nucleus (SCN). *PER1* has recently been shown to be associated with alcohol use, indicating that variation in *PER1 gene* mediated stress-induced drinking in animals and humans [41]. Molecular experiments in human *Per1* gene demonstrated a genotype specific increase in the transcription of *Per1* according to the concentration of glucocorticoids. This may point to a gene-stress interaction effect on the transcription of *Per1*, possibly resulting in different circadian organization under stressful conditions, which in turn may lead to self-medication with alcohol in those individuals.

Several limitations of the present study have to be considered. First, caution must be exercised in the interpretation of life stress, as it is difficult to separate the effect of environmental factors from genetic liability. Studies using genetically sensitive designs have indicated that many supposed environmental effects actually in part reflect genetic factors [42]. Thus, exposure to life events may be genetically mediated and the gene-environment interaction observed in this study might well be due to interactions between the *PER2* gene and other anonymous genes that were not identified (gene-gene interaction). Also in our study, the genotype groups differed slightly in their load of negative life events (see Table 1). However, as these differences are not statistically significant, and as the main effect of genotype is included in the regression model revealing the interaction effect, we do not assume the GxE effect to reflect merely a gene-environment-correlation. Second, another point of criticism may be the fact that the design of the present study is not completely longitudinal, with the interval of the life events between 15 and 19 years overlapping to a minor degree with the alcohol use assessed for the last 45 days. Even though we can assume that the majority of negative life events did not occur during this time period, but rather in the preceding four years, the direction of causality between life events, their interaction, and the genetic vulnerability on alcohol consumption at age 19 remains unclear.

Thirdly, the present findings have to be viewed in the light of a number of difficulties inherent in the detection of “true” gene-environment interactions. Major issues of criticism relate to the potential for multiple testing, low statistical power, and the lack of criteria for replication. Multiple testing has long been a serious problem in genetic research. The availability of datasets which afford large numbers of subdivisions (due to different ways of defining genotype and environmental characteristics) multiplies the potential of multiple testing by offering numerous additional possibilities for data mining [43]. Another difficulty in genetic association research is that many studies lack sufficient statistical power. Since statistical tests for examining interaction effects are less powerful than tests of main effects, this problem applies particularly to studies of GxE. The power to detect an interaction depends on a number of conditions, including the sample size as well as the distribution of genotypes and environmental exposures in the sample. Given the likely small effects of any single GxE and the associated risk of false positive results, this argues for the critical need for replication. However, differences in the measurement instruments, in assessing genotype, phenotype and environmental variables, between studies complicate to find comparable studies [44]. However, recent meta-analyses, e.g. by Karg et al. [39], have demonstrated that replication of GxE effects is actually possible, and that problems, such as low statistical power, could be overcome by using an at-risk group approach. In this line, the results reported here should be considered with caution regarding the sample size and the number of statistical tests performed. Hence, we do not exclude the possibility that the reported associations in the present study may reflect false-positive results (type 1 error), given that the discovery set is rather small and no replication or functional work is available. Also, it should be emphasized that the associations we observed are small in effect and would not hold up to stringent correction for multiple testing and thus should be regarded as preliminary.

In conclusion, in this study, we demonstrated an association of allelic variation in *PER2* gene with alcohol use in a sample of young adults from the community, replicating the findings of Spanagel et al. [11] in a patient sample. Carriers of the protective variant drank less frequently in total, and displayed fewer alcohol-related problems following exposure to stressful life events. Future research will have to disentangle the actual pathways by which stressful life events affect alcohol use in order to contribute to a better understanding of the underlying mechanisms. However, in light of the likely burden of multiple tests, the nature of the measures used and the nominal evidence of interaction, the GxE interaction reported here needs replication in independent samples, before firm conclusions can be drawn.
